# Accelerating Neuroimage Registration through Parallel Computation of Similarity Metric

**DOI:** 10.1371/journal.pone.0136718

**Published:** 2015-09-09

**Authors:** Yun-gang Luo, Ping Liu, Lin Shi, Yishan Luo, Lei Yi, Ang Li, Jing Qin, Pheng-Ann Heng, Defeng Wang

**Affiliations:** 1 Department of Stomatology, The Second Hospital of Jilin University, Changchun, 130041, Jilin, China; 2 Shenzhen Institutes of Advanced Technology, Chinese Academy of Sciences, Shenzhen, China; 3 Department of Medicine and Therapeutics, The Chinese University of Hong Kong, Hong Kong, China; 4 Chow Yuk Ho Technology Centre for Innovative Medicine, The Chinese University of Hong Kong, Hong Kong, China; 5 Research Center for Medical Image Computing, Department of Imaging and Interventional Radiology, The Chinese University of Hong Kong, Hong Kong, China; 6 Department of Radiology, The Second People's Hospital of Shenzhen, Shenzhen, China; 7 Department of Computer Science and Engineering, The Chinese University of Hong Kong, Hong Kong, China; 8 CUHK Shenzhen research institute, Shenzhen, China; 9 Department of Biomedical Engineering and Shun Hing Institute of Advanced Engineering, The Chinese University of Hong Kong, Hong Kong, China; Henry Jackson Foundation, UNITED STATES

## Abstract

Neuroimage registration is crucial for brain morphometric analysis and treatment efficacy evaluation. However, existing advanced registration algorithms such as FLIRT and ANTs are not efficient enough for clinical use. In this paper, a GPU implementation of FLIRT with the correlation ratio (CR) as the similarity metric and a GPU accelerated correlation coefficient (CC) calculation for the symmetric diffeomorphic registration of ANTs have been developed. The comparison with their corresponding original tools shows that our accelerated algorithms can greatly outperform the original algorithm in terms of computational efficiency. This paper demonstrates the great potential of applying these registration tools in clinical applications.

## Introduction

Neuroimage registration is useful for many neuroimage applications such as statistical quantification of human brain morphometry and computer-aided diagnosis. Neuroimage registration aims at aligning brain images in such a way that the same anatomical structure can correspond spatially by finding an optimal spatial mapping. Given two images, a reference image and a moving image to be registered, the registration problem is to find a transformation that minimizes the disparity by measuring the similarity between the transformed moving image and the reference image. Registration algorithms can be classified as linear and non-linear registration according to the type of transformations they permit.

Image registration often requires a long computational time due to the intensive computation of the transformation and similarity measure, which are considered as the bottleneck of image registration computational cost. Optimization algorithms in high-dimensional spaces are usually complex and time-consuming, which prolong data analysis and limit their use in clinical applications such as image-guided intervention. Using multi-core CPUs is one way to speed up the registration. On the other hand, GPUs have also been widely used for image processing applications including medical registration due to their inherent massive data parallelism [1][2][3][4]. Aiming at solving the computational bottleneck of image registration, some methods have been proposed to accelerate the similarity metric computation on GPU platform. Shams [5] accelerated 3D medical images registration by computing mutual information (MI) as the similarity metric on GPU. For registration algorithm based on MI, histogram computation is an essential component [6]. There have been several approaches to compute the histogram efficiently on the GPUs. Shams *et al*. [7] maintained a number of sub-histograms in global memory (due to shared memory volume restriction) and sum-up afterwards, or fit a few sub-histograms in shared memory but access the image volumes several times to cover the entire bins. Vetter *et al*. [8] presented a pre-processing sort to reduce the usage of shared memory and to guarantee coalesced write operations at the same time. For introduction of shared memory please refer [9]. They further facilitated the histogram collisions by allocating more counters for "fat bin" according to the intensity distribution obtained from a low cost one-dimensional histogram of the moving image during the pre-processing phase. For nonlinear registrations, Muyan-Ozcelik implemented a CUDA version of DEMONS registration algorithm [10]. James *et al*. introduced some GPU implementations of nonlinear registration such as the B-spline method [11]. And Mark *et al*. developed a open-source software tool NiftyReg for registration on GPU, which implemented a parallel version of the well-known free-form deformation algorithm using CUDA[12].

GPUs also bring opportunities for existing neuroimage registration tools such as FLIRT (FMRIB's Linear Image Registration Tool) [13][14][15]in FMRIB Software Library (FSL), which may be redeveloped and redesigned for a massive multi-processing architecture. FLIRT is demonstrated to be one of the highest robust and widely used linear image registration tools [15]. However, performing FLIRT on single CPU is time consuming. It took about one hour to register two brain volumetric images when it was first proposed in 2001 [14], and now, it still took a few minutes to perform affine registration based on our tests with workstations (2.53G Xeon processor 4GB RAM). To improve the efficiency, Chen [16] implemented the FLIRT algorithm with MI as the similarity metric on GPU. Since correlation ratio (CR) is a more widely used similarity metric in many applications [14], Li presented an accelerated FLIRT for volume image registration with CR as the similarity metric on GPUs [17].

Because linear registration algorithms are still inadequate to align brain structures perfectly, numerous nonlinear registration tools have been proposed, such as FNIRT (FMRIB’s nonlinear image registration tool) [15], SPM[18] and ANTs (Advanced Normalization Tools) [19][20][21]. Built on ITK framework, the ANTs open-source library provides a suite of tools on diffeomorphic normalization, image segmentation and template building, and it is commonly used in the medical image analysis field. According to a review paper [22], ANTs is one of the nonlinear registration methods with top performance. However, computational speed is still an issue for ANTs. It took about one and half hour to perform diffeomorphic registration for two 3D images (voxel resolutions: 256×256×128) on our tests with the workstation (2.53G Xeon processor, 4GB RAM). Because ANTs is rather complex, there is no hardware acceleration for it to the best of our knowledge.

The main contribution of this paper is to achieve fast implementation of the popularly used linear and non-linear image registration tools on the GPU platform. For FLIRT acceleration, the method was proposed in[17] is adopted, which just focused on the accelerating scheme on the linear registration tool FLIRT with CR as the similarity measure. This paper extends it with more experiments about neuroimage registration and further analysis about the accelerated algorithm. For accelerating CR calculation, an array for the reference image is first constructed, which is pre-sorted according to its intensity once on the GPU. Then CR is computed efficiently on the GPU kernel. In addition, we also implement a fast calculation of correlation coefficient (CC) on the GPU for the symmetric diffeomorphic registration of ANTs for non-linear neuroimage registration. For CC calculation on the GPU, 3D volume filtering with surface and texture memory is used. Finally the GPU implementations of both FLIRT and ANTs are extensively evaluated with datasets from public neuroimage databases. Our accelerated algorithms are compared with their original implementations in terms of both registration accuracy and computation efficiency.

## Preliminaries

### The FLIRT algorithm and Correlation ratio

FLIRT is a linear image registration tool freely available for precompiled binaries for non-commercial use [13]. Given a reference image *I*, and a moving image *J*, it uses a multistart, multiresolution global optimization method to find the affine transformation that minimizes the disparity between the reference image and the moving image [14]. It divides the process of searching for the best transformation at four different resolution levels: 8, 4, 2, 1mm. In practice, as in the original FLIRT, images are sub-sampled such that the voxel side-length becomes 1 mm according to their original physical dimensions of each voxel. The 2-, 4- and 8-mm sub-sampled volumes are obtained according to the isotropic 1-mm resolution volume.

For each search in each stage of the FLIRT algorithm, the transformed image *J*_*T*_ (*J*_*T*_ = *J* ∘ *T*) is obtained, and then the similarity metric between *J*_*T*_ and *I* is calculated for all the candidate transformations in the search space, which means that the computation of *J*_*T*_ and similarity metric is carried out iteratively.

The CR of two variables *P* and *Q* is defined as [23]:

$$\eta (P|Q)=\frac{Var[E(Q|P)]}{Var(Q)}=1-\frac{Var[Q-E(Q|P)]}{Var(Q)}$$ (1)

where *Var*[*E*(*Q* | *P*)] is the part of *Q* predicted by *P*, and *Var*(*Q*) is the total "energy" of *Q*. CR measures the functional dependence between *P* and *Q*. It takes on values between 0 (no functional dependence) and 1 (purely deterministic dependence).

Given a reference image *I*, and a transformed image *J*_*T*_, they may be seen as two discrete random variables, and their CR is computed as [23]:

$$\eta (J_{T}|I)=1-\frac{1}{N\sigma^{2}}\sum_{i}N_{i}\sigma_{i}^{2}$$ (2)

$$\sigma^{2}=\frac{1}{N}\sum_{\omega \in \Omega }J_{T}{\left(\omega \right)}^{2}-m^{2},\;m=\frac{1}{N}\sum_{\omega \in \Omega }J_{T}(\omega ),$$ (3)

$$\sigma_{i}{}^{2}=\frac{1}{N_{i}}\sum_{\omega \in \Omega_{i}}J_{T}{\left(\omega \right)}^{2}-m_{i}{}^{2},\;m_{i}=\frac{1}{N_{i}}\sum_{\omega \in \Omega_{i}}J_{T}(\omega )$$ (4)

where *σ* ^2^ and *m* are the variance and mean of voxels within Ω in *J*_*T*_, respectively. Ω denotes the overlapping region between the two images, and *N* is the number of voxels in Ω. Ω_*i*_ = {*ω* ∈ Ω, *I*(*ω*) = *i*} is the isointensity set of *I* and *N*_*i*_ is the number of voxels in Ω_*i*_. For image registration, the isointensity set of *I* is computed by the histogram bins of *I*, Ω_*i*_ = {*ω* ∈ *Bin*[*i*]}, in which *i* represents a gray-level of *I*. Compared with MI, the computation of CR does not require computing 2D-histograms of the images. The computational complexity of CR is *O*(*X*) O(n_x_) instead of O(n_x_n_y_) for a conventional algorithm for computing MI, *X* and *Y* being the number of gray levels in *I* and *J*_*T*_. Image registration with CR as the similarity metric is able to generate comparably accurate results and shows better robustness at low resolutions, compared with MI as similarity metric [14]. These advantages make CR especially useful for multi-resolution methods such as the FLIRT algorithm.

### Symmetric diffeomorphic image registration in ANTs with cross-correlation

The ANTs toolkit provides a hierarchy of transformations with adjustable levels of complexity, regularization, degrees of freedom and behavior as optimizers. Symmetric diffeomorphic image registration in ANTs is based on optimizing and integrating a time-varying velocity field. The map *ϕ*, over time, denotes a family of diffeomorphisms, *ϕ*(*x*,*t*): Ω×*t* → Ω, generated by integrating a time-dependent, smooth velocity field, $\upsilon \;:\;\Omega \times \text{t}\to R^{\text{d}}$, through the ordinary differential equation(o.d.e.): $\frac{d\phi (x,t)}{dt}=v(\phi (x,t),t),\phi (x,0)=x.$Ω is the image domain. To construct a symmetric implementation, the diffeomorphism, *ϕ*, in ANTs is decomposed into two components *ϕ*_1_ and *ϕ*_2_, according to composition property of diffeomorphisms, and the first integral is split into two time intervals reflecting the underlying optimized components of the velocity field. Avants *et al* define the variational optimization problem driven by CC based on the generalized standard Large Deformation Diffeomorphic Metric Matching (LDDMM) equation[21],

$$E_{CC}(I,J)=\underset{\phi_{1}}{\text{inf}}\;\underset{\phi_{2}}{\text{inf}}\;\int_{t=0}^{t=0.5}({\left|v_{1}(x,t)\right\|}_{L}^{2}+{\left\|v_{2}(x,t)\right\|}_{L}^{2})dt+\int_{\Omega }CC(I,J,x)d\Omega$$ (5)

where *v*(*x*, *t*) = *v*_1_(*x*, *t*) in *t* ∈ [0, 0.5],and *v*(*x*, *t*) = *v*_2_(*x*, 1 − *t*) in *t* ∈ [0.5, 1], and

$$CC(I_{T},J_{T},x)=\frac{\sum_{i}((I_{T}(x_{i})-m_{I_{T}}(x){\left(J_{T}(x_{i})-m_{J_{T}}(x)\right)}^{2}}{\sum_{i}(I_{T}(x_{i})-m_{I_{T}}{\left(x\right)}^{2}{\left(J_{T}(x_{i})-m_{J_{T}}(x)\right)}^{2}}$$ (6)

in which *I*_*T*_ = *I* ∘ *ϕ*_1_(*x*, 0.5), *J*_*T*_ = *J* ∘ *ϕ*_2_(*x*, 0.5) and $m_{I_{T}}(x)$ is mean value computed over a local (2×*r*+1)^*D*^ window centered at each position *x* in *I*_*T*_, where *r*, is the neighborhood radius and *D* is the image dimension.

Calculation of *CC* is time consuming when done natively, especially for 3D images. To generate *CC*(*I*_*T*_, *J*_*T*_, *x*) for each pixel, the following three intermediate values need to be calculated:

$$A(x)={\sum_{i}I_{T}(x_{i})}^{2}-(2\times m_{I_{T}}(x)\times \sum_{i}I_{T}(x_{i}))+n\times m_{I_{T}}{\left(x\right)}^{2}$$ (7)

$$B(x)={\sum_{i}J_{T}(x_{i})}^{2}-(2\times m_{J_{T}}(x)\times \sum_{i}J_{T}(x_{i}))+n\times m_{J_{T}}{\left(x\right)}^{2}$$ (8)

$$C(x)=\sum_{i}I_{1}(x_{i})J_{1}(x_{i})-m_{I_{T}}(x)\times \sum_{i}I_{T}(x_{i})-m_{J_{T}}(x)\times \sum_{i}J_{T}(x_{i})+n\times m_{I_{T}}(x)\times m_{J_{T}}(x)$$ (9)

where *n* = (2×*r*+1)^3^, is the number of voxels in the local window.

To get the above values, ∑ *I*_*T*_(*x*_*i*_), ∑ *J*_*T*_(*x*_*i*_), ∑ *I*_*T*_(*x*_*i*_)^2^, ∑ *J*_*T*_(*x*_*i*_)^2^, ∑ *I*_*T*_(*x*_*i*_)*J*_*T*_(*x*_*i*_) are calculated. When iterating through an image volume voxel by voxel, only a few of the voxels that are used to calculate these five values change, i.e. the voxels in the boundaries of the local window. The sum of most voxel in the local window can be reused to speed up the calculation.

## The Proposed Method

### GPU accelerated FLIRT with CR as similarity metric

**Fig 1:**
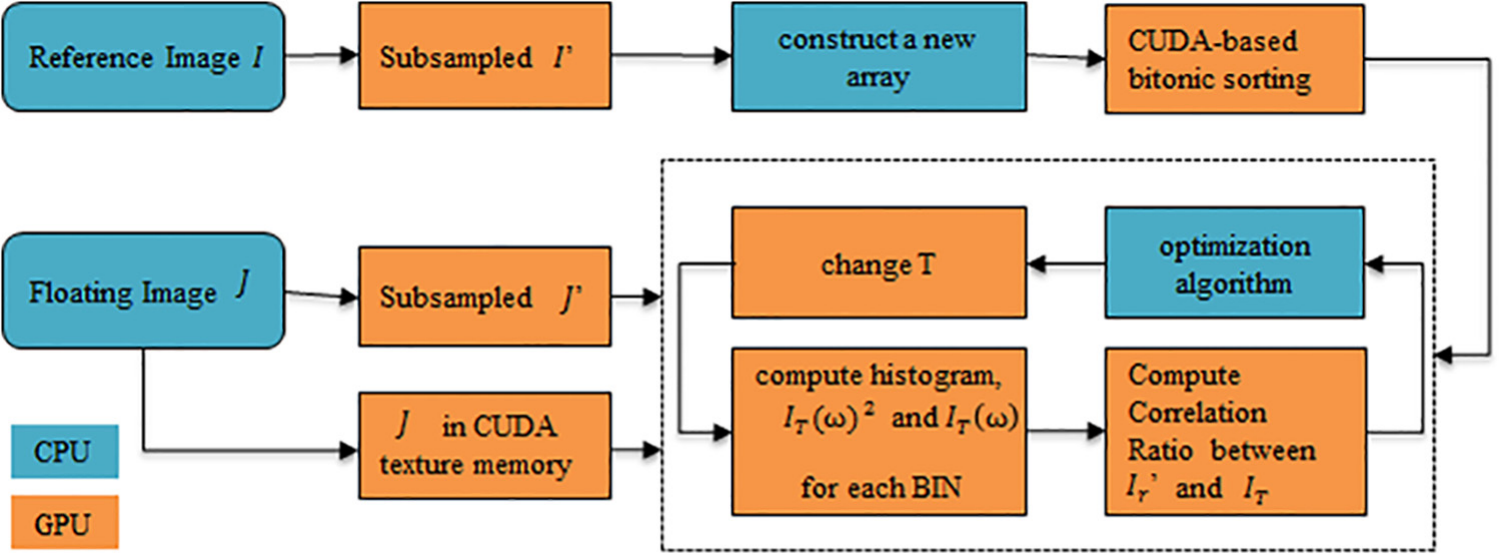
Flowchart of the GPU implementation for a stage of FLIRT.

An implementation of the original FLIRT framework specialized for GPUs was designed as in [17]. The sequential search was performed on the CPU, while all the computation-intensive processes such as re-sampling, transformation, interpolation of *J* and the computation of CR were performed on the GPU. An overview of the GPU implementation is shown in Fig 1. The process started with reading *I* and *J* to the global memory in the GPU, as well as binding *J* to the texture memory. *I* and *J* were re-sampled (if of sufficient quality) to an isotropic grid with voxel size 1-mm cubed in the GPU. The re-sampled 1-mm images were down-sampled three times to get the 2-, 4- and 8-mm images successively on the GPU. For each stage, by constructing an array, the subsampled reference image *I*' was sorted according to its intensity, and then a fast local optimization [5] was done to find the candidate transformations.

A pre-sorting of *I*' was required as a preprocessing step in each stage. An array *L* was constructed, each element of which contained a voxel’s coordinate and intensity. A CUDA-based sorting process [24][25] was applied to *L* with the intensity value as the key, guaranteeing that all the voxels belonging to the same histogram bin were distributed continuously.

$$L=\{(x,y,z,I)|I=I(x,y,z),(x,y,z)\in \Omega_{r}\}$$ (10)

The sorted array *L* was then passed to the CPU and a small routine was employed to mark the starting (and ending) index of each bin. The starting (and ending) index of each bin was kept in a marked array and transferred to the GPU’s global memory. The preprocessing step was executed once for each stage while the result might be used iteratively during the stage.

**Fig 2:**
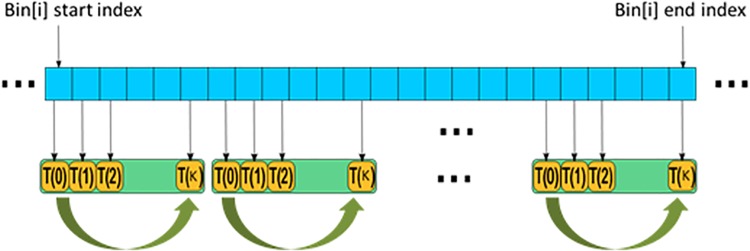
Sketch map of a block execution for computation of Bin[i].

When the sorted array *L* was available and the array saving the starting (and ending) index of each bin was transferred to the GPU, the CR computation kernel was called. Taking the formulas (2) to (3), $\sum_{\omega \in \Omega_{i}}I_{T}{\left(\omega \right)}^{2}$, $\sum_{\omega \in \Omega_{i}}I_{T}(\omega )$ (*ω* ∈ *Bin*[*i*]) and number of voxels within the mapped Ω_*i*_ were calculated to obtain $\sigma_{i}^{2}$ and *m*_*i*_ of those mapped voxels for this bin. The same number of thread blocks as that of the histogram bins was allocated so that each block would traverse the voxels from the start index to the end index for the bin on the sorted data, as shown in Fig 2 as in [17]. The pseudocode of the CR computation kernel is listed in algorithm in S1 Appendix. *K* (*K* = *WARP*_*SIZE*×*WARP*_*COUNT*) threads were allocated in each block, so the blocks need to execute (The end index of Bin[i]–the start index of Bin[i])/*K* or ((The end index of Bin[i]–the start index of Bin[i])/*K* + 1) times to process all the voxels from the start index to the end index for the corresponding histogram Bin[i].

After the number of voxels within Ω_*i*_, $\sum_{\omega \in \Omega_{i}}I_{T}{\left(\omega \right)}^{2}$ and $\sum_{\omega \in \Omega_{i}}I_{T}(\omega )$ for each bin were obtained, a CR sum kernel based on the reduction algorithm in CUDA was called for summing those results of each bin to get CR.

### GPU-accelerated CC calculation in ANTs

Symmetric diffeomorphic image registration in ANTs was implemented based on ITK framework, which provides lots of iterators to traverse an image. The iterator operator sequentially traverses an image volume, leading to a long calculation time. To explore the possibility of its hardware acceleration, the time consuming calculation of CC was done in parallel according to Eq 6, i. e., each voxel is calculated separately and independently, therefore great time saving is expected.

Based on the source code of ANTs, *I*_*T*_ and *J*_*T*_ were obtained from ITK image classes for the reference image and the moving image, respectively, transferred to the GPU global memory, and bound to two 3D texture memories. Five 3D surface memories were defined and $m_{I_{T}}$, $m_{J_{T}}$, *A*, *B* and *C* were bound to them for calculating CC, respectively, as surface memories in CUDA program environment can both read and write. Once the five values were calculated in the GPU, they were copied to the corresponding CPU memory allocated to store them for the subsequent calculation of ANTs.

$m_{I_{T}}$, and $m_{J_{T}}$ were obtained by mean filter of *I*_*T*_ and *J*_*T*_, respectively. They were implemented by volume filtering. The offsets of voxels in the local window respect to the center voxel were obtained in the CPU and copied to constant memory of the GPU. $m_{I_{T}}$, $m_{J_{T}}$, *A*, *B* and *C* were computed according to Eqs 7–9 in a GPU kernel. Each voxel was assigned to one thread, as the calculation is independent. Full description of the kernel is given in algorithm in S2 Appendix.

## Experimental Results and Discussion

### Datasets

For repeatable comparison, all datasets were downloaded from public databases. 4 datasets (NewHaven_b: Dataset 1, Bangor: Dataset 2, Oxford: Dataset 3, PaloAlto: Dataset 4) were downloaded from [26], and 10 subjects were chosen randomly in each datasets. In addition, the dataset including 18 subjects from IBSR (Dataset 5) with labeled data was also used [27]. For each dataset, a randomly selected image was chosen as the reference image, and the other images were considered as moving images and registered to the reference image.

### Implementation environment

A GPU version of FLIRT with CR as the cost function was implemented based on NVIDIA’s GPGPU programming environment, CUDA v6.5. The accelerated FLIRT runs on a workstation with Intel(R) Xeon(R) CPU W3505 @2.53GHz (RAM 4.00GB) and GTX 680 (Workstation 1). The detailed configuration of GTX 680 is listed in Table 1. The original FLIRT in FSL run with CentOS 7 installed on the same workstation.

**Table 1:** The hardware specification of theworkstastions with GTX680 and GTX 660, respectively.

Device specification (GPU)	GTX680	GTX660
Number of multiprocessors	8	2
Number of cores per multi-processor	192	192
Total amount of global memory	4096 MBytes	512 MBytes
Total number of registers available per block	65536 bytes	65536 bytes
Total amount of shared memory per block	49152 bytes	49152 bytes
Total amount of constant memory	65536 bytes	65536 bytes
Maximum number of threads per block	1024	1024
Warp size	32	32
CPU specification	Intel(R) Xeon(R) CPU W3505 @2.53GHz (RAM 4.00GB)	Intel(R) Core(TM) i5-3470S CPU @2.90GHz (RAM 8.00GB)

The original ANTs and the accelerated ANTs with CC calculation on GPU also run on (Workstation 1). To exam the impact of hardware configuration, the original ANTs and the accelerated ANTs with CC calculation on GPU run on another workstation with Intel(R) Core(TM) i5-3470S CPU @2.90GHz (Memory 8.00GB) and GTX 660 (Workstation 2) as well. The software can be downloaded through the website: http://figshare.com/articles/GPU_accelerated_FLIRT_and_ANTs_zip/1501449.

### Registration with FLIRT and the accelerated one

**Fig 3:**
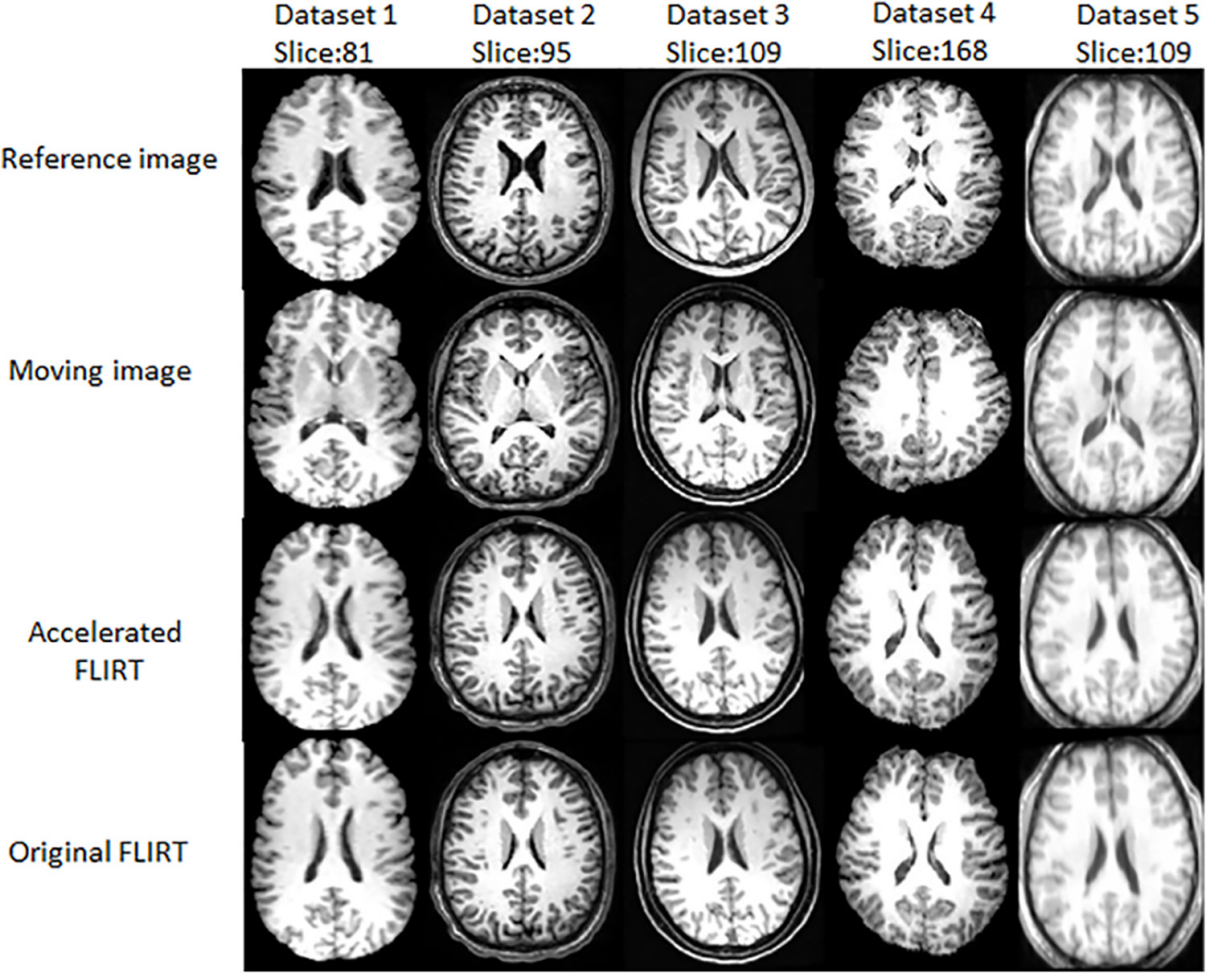
Registered results obtained by the accelerated FLIRT with CR as similarity metric and original FLIRT with CR as similarity metric.

For visual comparison, the slices of the registered results of the 5 datasets by original FLIRT and the accelerated FLIRT with CR as similarity measure are given in Fig 3. The slices with distinct features are displayed for easy visual comparison. Images in the first row are the reference images, images in the middle row are the results obtained by the accelerated FLIRT with CR as similarity measure, while those in the third row are results obtained from the original FLIRT. Fig 3 shows registered results of the accelerated FLIRT appear the same as those of the original tool.

The total runtimes of the accelerated FLIRT and original FLIRT for the 5 datasets are listed in Table 2. Each subject in each dataset was registered to the reference image 3 times. The mean runtime of the subjects in the same dataset is considered as the runtime of that dataset. On Workstation 1, the accelerated FLIRT took less than 30 seconds for all datasets, while the original FLIRT took about 1–2 minutes and scaled linearly with the number of voxels. Table 2 shows that the runtime of our accelerated FLIRT doesn’t increase clearly as the number of voxels of images increase. We get 4 times speedup with the accelerated FLIRT, while 18 times is achieved in [17]. This is because not only the hardware configurations of our workstation is different from that in [17], but also the operating system. They used Nvidia Tesla C2075, which is better than our GTX 680. On the other hand, as original FLIRT can only run in a virtual machine in windows system as in our case, it would take more time than in a linux system as in [17].

**Table 2:** Mean runtimes for registration of images from the 5 datasets of the original FLIRT and the accelerated FLIRT with Workstation 1. (Time: seconds).

Dataset time (s)	1	2	3	4	5
Dimensions (Width*Height*Depth)	131×179×137	162×215×157	176×192×192	256×124×256	256×256×128
Size of voxels	3212513	5468310	6488064	8126464	8088608
Original FLIRT	46.9	63.74	75.66	95.51	118.29
Accelerated FLIRT	13.72	23.17	25.05	25.54	24.56
Total speedup	3.4	2.8	3	3.7	4.8

The overall performance of a registration algorithm depends on the efficiency of the optimization strategy decided by the iterations required by the algorithm to converge. If a single computation of the similarity metric is considered in one iteration, the total runtime depends on the number of iterations and effectiveness in computing the similarity metric. For FLIRT, it uses a multistart, multiresolution global optimization method to eliminate the occurrence of gross misalignments in affine image registration. Its search space is relatively stable, then the key factor of running time of FLIRT is the time each iteration takes. As CR is calculated in each iteration, the runtime scales with the image sizes when it is calculated in the original FLIRT on CPU. While for the accelerated FLIRT with CR as similarity metric, the total runtime mainly depends on runtime of the sorting process and the time of CR calculation. When calculating CR on GPU, the voxels are grouped according to histogram bin, voxels in the same bin are calculated in parallel, i.e. voxels within the mapped Ω_*i*_, $\sum_{\omega \in \Omega_{i}}I_{T}{\left(\omega \right)}^{2}$, $\sum_{\omega \in \Omega_{i}}I_{T}(\omega )$ (*ω* ∈ *Bin*[*i*]) for each bin are calculated in a CR computation kernel at the same time to obtain $\sigma_{i}^{\;2}$ and *m*_*i*_ for that bin. Then another CR sum kernel is called to sum the above $\sum_{\omega \in \Omega_{i}}I_{T}{\left(\omega \right)}^{2}$, $\sum_{\omega \in \Omega_{i}}I_{T}(\omega )$ (*ω* ∈ *Bin*[*i*]) of all bins to get CR. Due to the above parallel computation of CR, CR calculation consume just increases slightly as the sizes of images increase. Meanwhile, the sorting time is random according to the reference image. Because the time increase due to the reference image size is not very obvious, in addition to the affection of sorting, the total runtime doesn’t scale with the image size clearly.

For GPU kernels, when called, they are executed N times in parallel by N different CUDA threads. And the threads schedule is according to the kernel parameters: grid size, block size and shared memory size. The block size of the CR computation kernel was set to WARP_SIZE*WARP_COUNT according to the configuration of the GPU used, being 32*8 for GTX 680. The grid size was set to the number of bins, which was 256/n used at resolution n (n = 8, 4, 2, 1). Because the number of bins for each stage of the native FLIRT algorithm is fixed, the runtime of CR computation kernel depends on the time required for traversing voxels in each bin, which is decided by the size of the reference image. The shared memory size for each block, local registers for each thread took by the GPU kernels were fixed and far less than the resources GTX 680 may afford, so the limitation of the GPU implementation comes from the global memory size and texture memory size. The original and the 1-, 2-, 4- and 8-mm moving and reference images were all kept in the global memory, as well as the sorted array L.

### Registration with ANTs and the accelerated one

**Fig 4:**
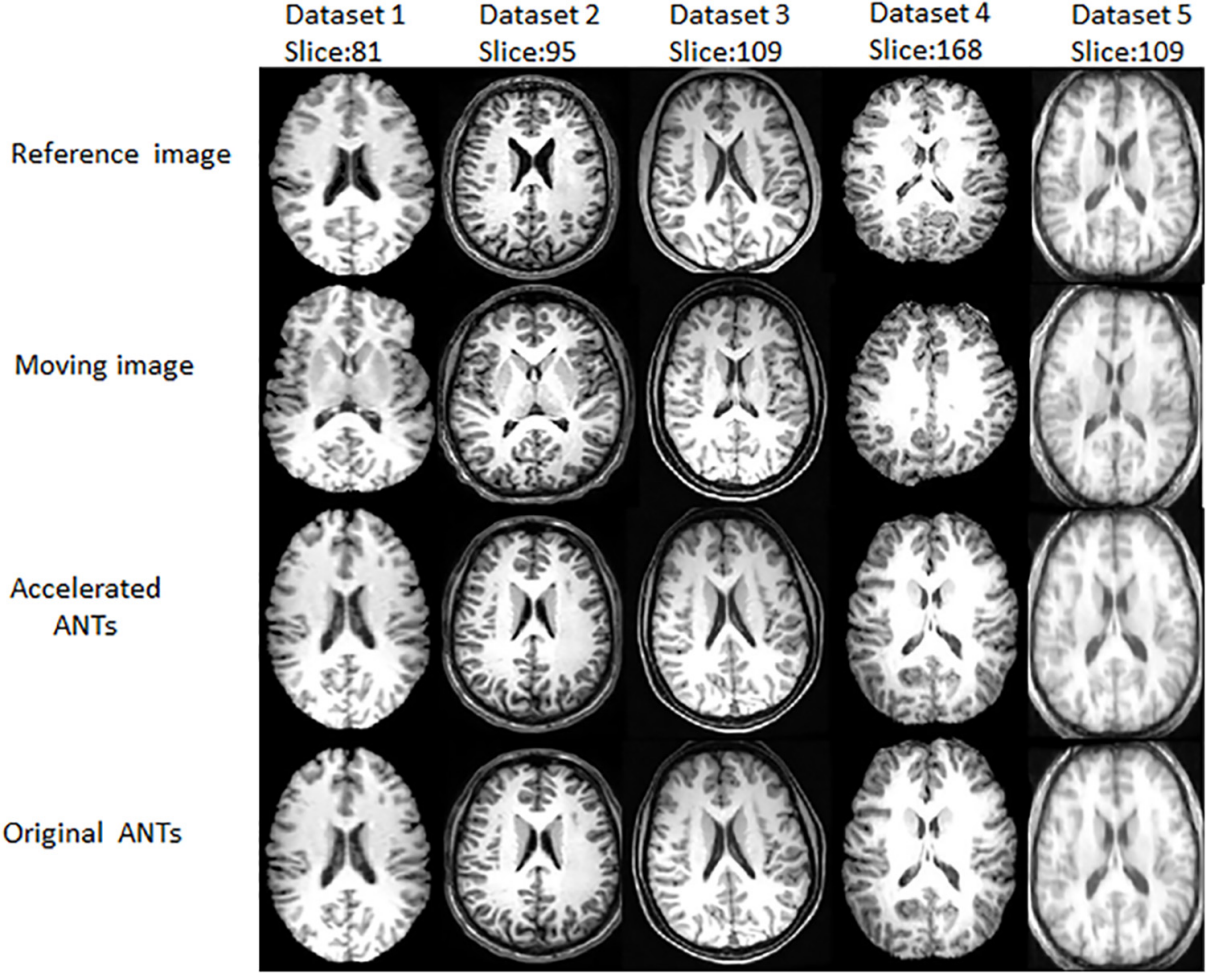
Registered results obtained by the GPU accelerated CC calculation for the symmetric diffeomorphic registration of ANTs and the original ANTs.

The slices of the registered results of the original ANTs and the accelerated ANTs with CC calculation on GPU are displayed in Fig 4. The images in the second row look almost the same as those in the third row, showing the accuracy of the acceleration algorithm. Figs 3 and 4 also show ANTs, as nonlinear registration tools, performs better than the linear FLIRT, as images registered with ANTs are more similar to the reference images than those with FLIRT.

**Fig 5:**
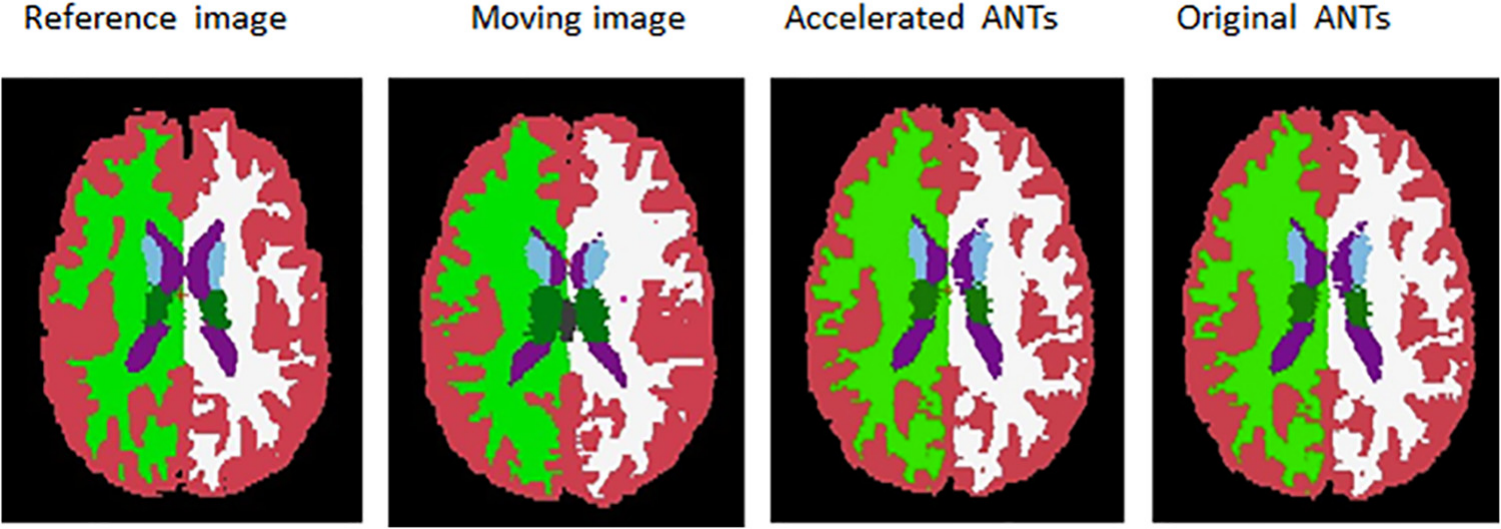
Registered results of region labeled images provided by the IBSR by the GPU accelerated CC calculation for the symmetric diffeomorphic registration of ANTs and the original ANTs.

To qualitatively evaluate the nonlinear registration accuracy, for the first four datasets without ground truth label images, mean absolute difference (MAD) between the registered results obtained by the original tools and the reference images, and MAD between the results achieved by the accelerated implementations and the same reference images were calculated and compared in Fig 5. The red bars show the MADs between the registered results using original tools and the reference image, while the blue bars are the MADs between the registered results using the accelerated implementations and the reference image. For the dataset IBSR, for each pair of registration, the resulting transformation was also applied to transform the brain structure label images provided by IBSR. The transformed label images were compared with the ground truth labels of the reference image in terms of structure volume overlap [28]. The structure volume overlap is measured with the Jaccard index:

$$J_{A,B}=\frac{\left|A\cap B\right|}{\left|A\cup B\right|}$$ (11)

**Fig 6:**
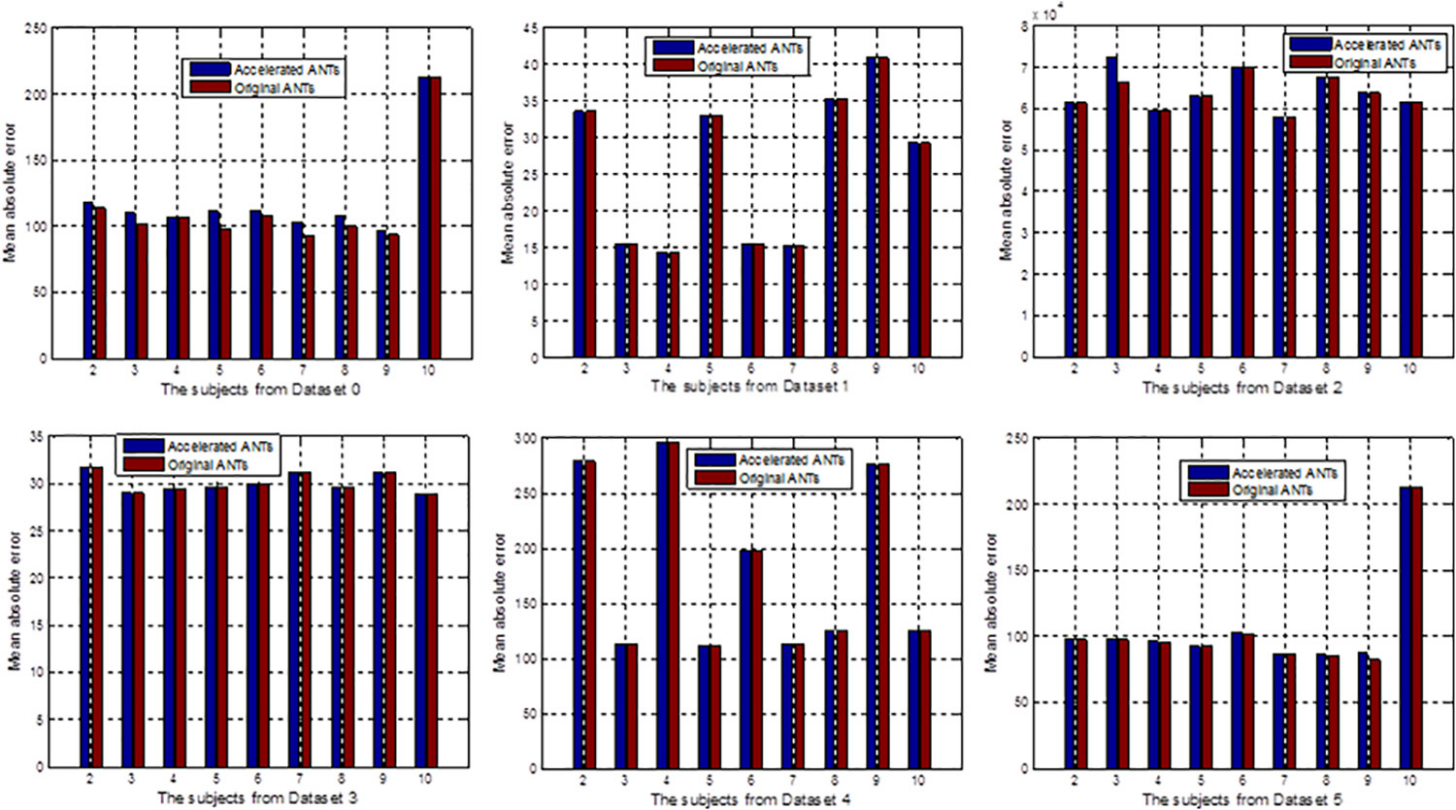
Mean absolute differences between the registered results obtained by original ANTs and the reference image, and those between the reference and the results obtained by the accelerated ANTs with CC calculation on GPU.

The Jaccard indice are almost the same between accelerated ANTs and original ANTs. Figs 5 and 6 also shows that the accelerated ANTs with CC calculation on GPU is nearly as accurate as the original ANTs. The results are slightly different because NVIDIA GPUs differ from the x86 architecture in that rounding modes are encoded within each floating point instruction instead of dynamically using a floating point control word. And sometimes the results by the accelerated ANTs are even slightly more accurate thanks to the fused multiply-add operator on GPU.

For the symmetric diffeomorphic image registration in ANTs, the total runtimes of original ANTs and that with GPU accelerated CC calculation are compared and given in Tables 3 and 4. From Table 3, it can be seen that the accelerated implementation achieved about 113.7 times speed up for CC calculation, and about 2 times speed up in total for all the datasets on Workstation 1. On Workstation 2, the accelerated implementation achieved about 16.7 times speed up for CC calculation, and about 2.2 times speed up in total, as listed in Table 4. Tables 3 and 4 shows the better the graphic card the faster CC calculation is, as the mean speed up times for CC calculation on GTX 680 is about 6.8 of that on GTX 660. GTX 680 has 8 multiprocessors, while GTX 660 only has 2 multiprocessors. However, Workstation 2 is equipment with a more powerful CPU than Workstation 1, so there is not much difference in the overall speed ups.

**Table 3:** Mean runtimes and speedups for registration of images from the 5 datasets of the original ANTs and the accelerated ANTs with Workstation 1 (Time: seconds).

Dataset time (s)	1	2	3	4	5
Dimensions (Width*Height*Depth)	131×179×137	162×215×157	176×192×192	256×124×256	256×256×128
Size	3212513	5468310	6488064	8126464	8088608
Time for CC in Original ANTs	1050.79	1699.62	2002.19	3170.54	3461.48
Total time for Original ANTs	1972.19	3062.61	3526.72	5023.48	6197.47
Percent of time for CC	53%	55%	57%	63%	56%
Time for CC in Accelerated ANTs	11.46	17.45	19.79	28.87	29.27
Total time for Accelerated ANTs	934.81	1398.15	1566.96	3713.58	3452
Speedup of CC	91.69	97.4	101.17	109.82	118.26
Total Speedup	2.11	2.19	2.25	1.35	1.81

**Table 4:** Mean runtimes and speedups for registration of images from the 5 datasets of the original ANTs and the accelerated ANTs with Workstation 2. (Time: seconds).

Dataset time (s)	1	2	3	4	5
Dimensions (Width*Height*Depth)	131×179×137	162×215×157	176×192×192	256×124×256	256×256×128
Size (voxels)	3212513	5468310	6488064	8126464	8088608
Time for CC in Original ANTs	591.31	966.86	1126.05	1742.72	1982.59
Total time for Original ANTs	996.81	1616.6	1838.79	2627.15	3039.34
Percent of time for CC	59%	60%	61%	66%	62%
Time for CC in Accelerated ANTs	39.37	60.83	72.76	109.88	107.26
Total time for Accelerated ANTs	443.37	676.57	781.64	1011.11	1108.66
Speedup of CC	15.02	15.9	15.48	15.86	18.48
Total Speedup	2.25	2.38	2.35	2.6	2.74

For the two graphic cards, the detailed configurations of the CC computation kernel are the same. The block size was set to (8, 8, 1), with each thread calculating a voxel, while the grid size is set according to the image volume size and the block size, as that in the example of volume filtering in the CUDA SDK. As listed in algorithm in S2 Appendix, *I*_*T*_ and *J*_*T*_ are bound to texture memory, and $m_{I_{T}}$, $m_{J_{T}}$, *A*, *B* and *C* are bound to surface memory, so the limitation of implementation is the texture memory size GTX 680 and GTX 660 may afford. The offsets of voxels in the local window respect to the center voxel are kept in the GPU constant memory. The size of the offsets depends on the neighborhood radius *r* set for CC calculation, now *r* can be set to a maximum 8 as the constant memory sizes of GTX 680 and 660 are both 65536 bytes, while *r* is usually set 5 according to [20]. In the above experiments, all r are set to 5.

## Conclusions

A GPU implementation of FLIRT with CR as the similarity metric is developed. On the GPU device a pre-sorting on the reference image is computed once. Then CR, the default cost function of FLIRT, is calculated efficiently on the GPU without read-write conflict. A GPU accelerated CC calculation for the symmetric diffeomorphic registration of ANTs is also implemented. Comparisons with the corresponding original tools have shown the advantages of the proposed methods in terms of computational efficiency and accuracy. The proposed method improved the usefulness of the original tools for clinical applications. Our future work includes further accelerating deformable registration algorithms, such as the whole algorithms in the ANTs based on the proposed method.

## Supporting Information

S1 Appendix (DOCX)

S2 Appendix (DOCX)
