# Supplementary material for: Accelerating Neuroimage Registration through Parallel Computation of Similarity Metric
Source: PLoS One. 2015 Sep 9;10(9):e0136718. doi: 10.1371/journal.pone.0136718 (PMC4564209; doi:10.1371/journal.pone.0136718)
Supplement: S1 Appendix — (DOCX) [file pone.0136718.s001.docx]

S1 Appendix. Algorithm of the CR computation kernel

| **Algorithm 1** | the CR computation kernel |
| --- | --- |
| blockSize ← blockDim.x | |
| tid ← threadIdx.x | |
| startIndex ← dBlockPivots[currentBin] | |
| endIndex ← dBlockPivots[currentBin+1] | |
| blockSize ← blockDim.x  tid ← threadIdx.x  startIndex ← dBlockPivots[currentBin]  endIndex ← dBlockPivots[currentBin+1]  val ← 0 // $I_{T}\left( \omega\right)$  val2 ← 0 //$I_{T}{(\omega)}^{2}$  N_currentBin_ ← 0 // number of voxels in the current Bin  **For** (index = startIndex+tid; index < endIndex; index += blockSize) **do**  Fetch the coordination of dRefSortedData and shift to the mass center  transform to the moving volume  Shift to the moving volume's coordinate  **if** the coordinate is in the domain of the moving volume **then**  //accumulate $I_{T}\left( \omega\right)$, $I_{T}{(\omega)}^{2}$, and number of voxels in the current Bin  srcIntensity ← tex3D(tSrcVolData, srcCoord) //read from 3D texture  val ← srcIntensity + val  val2 ← srcIntensity $\times$ srcIntensity + val2  NcurrentBin ← NcurrentBin + 1  **end if**  **end for**  sval[tid] ← val  sval2[tid] ← val2  sNcurrentBin[tid] ← NcurrentBin  synchronization  sum up all the threads to get the number of voxels in this bin, $\sum_{\omega\in\Omega_{i}} I_{T}{(\omega)}^{2}$and$\sum_{\omega\in\Omega_{i}} I_{T}\left( \omega\right)$ ($\omega\in Bin[i]$) | |
|  | |
