# Supplementary material for: Accelerating Neuroimage Registration through Parallel Computation of Similarity Metric
Source: PLoS One. 2015 Sep 9;10(9):e0136718. doi: 10.1371/journal.pone.0136718 (PMC4564209; doi:10.1371/journal.pone.0136718)
Supplement: S2 Appendix — (DOCX) [file pone.0136718.s002.docx]

S2 Appendix. Algorithm of the CC computation kernel

| **Algorithm 2** | the CC computation kernel |
| --- | --- |
| Get voxel index (x,y,z) in 3D from thread ID, block ID, and block Dim  **if** (x,y,x) is outside of the volume index then  return  **end if**  cura,curb,a,b,suma2,sumb2,sumab,suma,sumb,count $\leftarrow$ 0  cura $\leftarrow$ Texture fetch at (x,y,z) from the texture for $I_{1}$  curb $\leftarrow$ Texture fetch at (x,y,z) from the texture for $J_{1}$  **for** i $\leftarrow$ 0, n-1 do  the index (x’,y’,z’) of the ith voxel in the local window $\leftarrow$ (x,y,z) + the ith voxel’s offset  **if** (x’,y’,z’) is inside of the volume index then  a $\leftarrow$ Texture fetch at (x’,y’,z’) from the texture for $I_{1}$  b $\leftarrow$ Texture fetch at (x’,y’,z’) from the texture for $J_{1}$  suma2 $\leftarrow$ suma2 + a$\times$a  sumb2$\leftarrow$ sumb2 + b$\times$b  suma $\leftarrow$ suma + a | |
| sumb $\leftarrow$ sumb + b | |
| count $\leftarrow$ count +1  **end if**  **end for**  **if** count > 0 **then** $\bar{I}(x,y,z)$ $\leftarrow$   suma / count $\bar{J}(x,y,z)$ $\leftarrow$   sumb / count $A(x,y,z)$ $\leftarrow$   suma2 - 2$\times$ $\bar{I}(x,y,z)$ $\times$suma + count $\times$ $\bar{I}(x,y,z)$ $\times$ $\bar{I}(x,y,z)$ $B(x,y,z)$ $\leftarrow$    sumb2 - 2$\times$ $\bar{J}(x,y,z)$ $\times$sumb + count $\times$ $\bar{J}(x,y,z)$ $\times$ $\bar{J}(x,y,z)$ $C(x,y,z)$ $\leftarrow$    sumab -$\bar{I}(x,y,z)$ $\times$suma - $\bar{J}(x,y,z)$ $\times$sumb + count $\times$ $\bar{I}(x,y,z)$ $\times$ $\bar{J}(x,y,z)$ $\bar{I}(x,y,z)$ $\leftarrow$     cura - suma / count $\bar{J}(x,y,z)$ $\leftarrow$   curb - sumb / count  write $A(x,y,z)$, $B(x,y,z)$, $C(x,y,z)$, $\bar{I}(x,y,z)$, $\bar{J}(x,y,z)$to 5 surface textures, respectively  **end if** | |
|  | |
